# Notes and Comments

**Published:** 1899-01

**Authors:** 


### Notes and Comments.[1]

Some of the Dangers in Fruit.—In writing upon this subject the *Family Doctor* says we have heard much of late about appendicitis, and a great many people who are fond of fruit, and who need it to keep in a healthy condition, discard its use through fear of being attacked by it. As a fact, the disease is not any more common than it used to be, and it is foolish for persons to deny themselves the pleasure of eating fruit through fear of microbes or appendicitis because, perhaps, one in a million persons happens to get a seed in the "appendix." There are, however, precautions which it would always be well to take; for example, all fruit with skins on should be washed and peeled before being eaten, especially fruit exposed in the street, and where dust and flies can have access to it. Few are aware of the danger of food contamination by flies. They are great scavengers, and are not at all discriminating as to what they eat nor where they settle. They pass at one bound from an infectious carcass, a foul ulcer, or a mass of filth to the apple, pear, or peach, and with dirty feet and proboscis run over it and contaminate it. Hence all fruit should be first washed and dried, and then peeled.

Artistic Dentistry.—Under this head the *Dental Weekly* says many dental operations might be grouped, but there is one which is more æsthetical than useful, but which is appreciated by patients. It is the shortening of elongated front teeth by grinding them to conform to a more natural expression. The points of cuspids frequently present the appearance of tusks, because of their length. The removal of the points with a stone adds much to the appearance of the mouth. Frequently a central or a lateral incisor, from some cause, will elongate and mar the symmetry of a dental arch. A little grinding will add much to looks. In some cases the point of the outer cusp of a bicuspid can be removed with good effect.

[1] The assistant editor solicits contributions for this department,—new methods, new remedies and formulas, or any short practical note which may prove of value to the practitioner or student. Address 1338 Walnut Street, Philadelphia.

A Method of working Cement.—To insure a smooth, hard cement filling in proximal surfaces, particularly where the labial and lingual walls are to be restored, pass a thin piece of mica or celluloid matrix, it first having been slightly oiled, between the teeth, and, after introducing the cement into the cavity, press the mica or celluloid matrix firmly over the cavity and hold in this position for a few moments. The pressure makes a more solid filling. The oil prevents the cement sticking to the matrix, and the matrix gives the proper space between the teeth.—J. A. C., in *Dental Weekly.*

---

A Substitute for Rubber Gloves.—We all know that in warm weather rubber gloves cannot be worn with comfort while engaged in prosthetic work; to subserve the same purpose the following suggestion for an ointment is taken from the *Medical Brief:* "In the warm days that are now before us, when a rubber glove cannot be worn with comfort while engaged in prosthetic work, an ointment of honey for the hands will subserve the same purpose. It holds the dirt in suspension and dissolves very quickly when immersed in water, leaving the hands soft and clean. Take clarified honey and rose water, of each one pint, listerine two ounces. Mix and bottle. For winter use, add two or three ounces of glycerin."

---

Appointments of Dentists for the Army and Navy.—We take the following from our British friend, *The Dentist*, published in London: America is making a new departure. There has been introduced into the United States Congress a bill providing for the appointment of a brigade dentist for each brigade, with the title of major, and one for each regiment with the title of captain. The idea can be commended to our naval authorities. Why should our blue-jackets, if they require skilled dental attention, have to wait until they are on shore? Our soldiers are generally within reach of experienced dentists; but our jack-tars on board ship have to be contented with the services of the ship's doctor—who may not know much about teeth—or he must wait until he gets home again. Now that dentistry is beginning to have proper respect paid it, perhaps some one will take up this point.

